# Quadriceps Muscles O_2_ Extraction and EMG Breakpoints during a Ramp Incremental Test

**DOI:** 10.3389/fphys.2017.00686

**Published:** 2017-09-19

**Authors:** Danilo Iannetta, Ahmad Qahtani, Guillaume Y. Millet, Juan M. Murias

**Affiliations:** Faculty of Kinesiology, University of Calgary Calgary, AB, Canada

**Keywords:** [HHb] breakpoint, electromyography, quadriceps muscle activity, exercise intensity boundaries, ramp incremental test

## Abstract

Muscle deoxygenated breakpoint ([HHb]*BP*) has been found to be associated with other indices of exercise tolerance in the vastus lateralis (VL) muscle but not in the vastus medialis (VM) and rectus femoris (RF).

**Purpose:** To investigate whether the [HHb]*BP* occurs also in the VM and RF muscles and whether or not it is associated with other physiological indices of exercise tolerance, such as the EMG threshold (EMG_t_) and the respiratory compensation point (RCP).

**Methods:** Twelve young endurance trained participants performed maximal ramp incremental (RI) cycling tests (25–30 W·min^−1^ increments). Muscle oxygen extraction and activity as well as ventilatory and gas exchange parameters were measured. After accounting for the mean response time, the oxygen uptake ($\overset{\cdot }{\text{V}}$O_2_) corresponding to the RCP, [HHb]*BP*, and the EMG_t_ was determined.

**Results:** Peak power output (PO_peak_) was 359 ± 48 W. Maximal oxygen consumption ($\overset{\cdot }{\text{V}}$O_2max_) was 3.87 ± 0.46 L·min^−1^. The $\overset{\cdot }{\text{V}}$O_2_ at the RCP was 3.39 ± 0.41 L·min^−1^. The $\overset{\cdot }{\text{V}}$O_2_ (L·min^−1^) corresponding to the [HHb]*BP* and EMG_t_ were: 3.49 ± 0.46 and 3.40 ± 0.44; 3.44 ± 0.61 and 3.43 ± 0.49; 3.59 ± 0.52, and 3.48 ± 0.46 for VL, VM, and RF, respectively. Pearson's correlation between these thresholds ranged from 0.90 to 0.97 (*P* < 0.05). No difference was found for the absolute $\overset{\cdot }{\text{V}}$O_2_ and the normalized PO (%) at which the thresholds occurred in all three muscles investigated (*P* > 0.05). Although in eight out of 12 participants, the [HHb]*BP* in the RF led to a steeper increase instead of leading to a plateau-like response as observed in the VL and VM, the $\overset{\cdot }{\text{V}}$O_2_ at the breakpoints still coincided with that at the RCP.

**Conclusions:** This study demonstrated that local indices of exercise tolerance derived from different portions of the quadriceps are not different to the systemic index of the RCP.

## Introduction

When performing a ramp incremental (RI) cycling test to the limit of tolerance, a linear increase in cardiac output ($\overset{\cdot }{\text{Q}}$) occurs in order to meet the increasing oxygen (O_2_) demand due to the increased muscular work (Astrand et al., 1964). This linear increase in $\overset{\cdot }{\text{Q}}$ for a given increase in metabolic demand during a RI test is accompanied by a hyperbolic increase in arteriovenous O_2_ difference (a-v*O*_2diff_) to support the O_2_ demand until the individual's maximal O_2_ consumption ($\overset{\cdot }{\text{V}}$O_2max_) is achieved and the exercise can no longer be sustained.

Despite these systemic adjustments for $\overset{\cdot }{\text{Q}}$ and a-v*O*_2diff_, the profile of the near infrared spectroscopy (NIRS)-derived O_2_ extraction ([HHb]) of the vastus lateralis muscle (VL) during a RI test to exhaustion generally shows a linear increase up to a point where this increase is attenuated and a plateau-like response is manifested (Spencer et al., 2012; Murias et al., 2013a,b). This attenuation in the [HHb] signal is suggestive of a lower reliance on O_2_ extraction at the level of the working muscles (specifically the muscle portion interrogated by the NIRS probe), despite the O_2_ consumption ($\overset{\cdot }{\text{V}}$O_2_) continuing to rise along with the increments in power output (PO). The point at which the linearity of the increase in the [HHb] signal is disrupted has been described as a breakpoint ([HHb]*BP*) (Spencer et al., 2012), and it has been shown to occur at a metabolic rate (i.e., $\overset{\cdot }{\text{V}}$O_2_) similar to the respiratory compensation point (RCP) (Murias et al., 2013b; Fontana et al., 2015; Keir et al., 2015; Iannetta et al., 2017) and the electromyography (EMG) threshold (EMG_t_) (Hug et al., 2003a,b; Osawa et al., 2011; Racinais et al., 2014). Interestingly, Boone et al. (2016) recently suggested that, at least in the vastus lateralis (VL) muscle, a cascade of events may occur as the EMG_t_ was found to precede the RCP which in turn preceded the [HHb]*BP*.

Given that the occurrence of the [HHb]*BP* during RI tests has been only examined in the VL muscle (Spencer et al., 2012; Murias et al., 2013a,b; Fontana et al., 2015; Boone et al., 2016; Iannetta et al., 2017), it is unknown whether the same behavior in the dynamic adjustment of the [HHb] signal would be observed in other muscles involved in cycling, such as the vastus medialis (VM) and the rectus femoris (RF). This is an important issue that needs to be addressed as it is likely that due to differences in perfusive and diffusive capacity (Poole et al., 2013), muscle fiber composition (Johnson et al., 1973), patterns of muscle recruitment (Chin et al., 2011) as well as differences in aerobic fitness (Boone et al., 2009), regional heterogeneities in muscle blood flow (Q_m_) and muscle $\overset{\cdot }{\text{V}}$O_2_ ($\overset{\cdot }{\text{V}}$O_2m_), and activation (Hug, 2010; Okushima et al., 2015) may affect the correspondence between the [HHb]*BP* and EMG_t_ with the RCP. In fact, Chin et al. (2011) have shown that during RI cycling test to exhaustion, VL and VM [HHb] had similar profiles which in turn substantially differed from that of the RF. Interestingly, all three profiles of [HHb] were described to resemble those of the EMG, suggesting a correspondence between oxygen extraction and muscle activation. However, a sigmoidal model fit was used in that study, and thus no relationship on the basis of $\overset{\cdot }{\text{V}}$O_2_ was established between the [HHb]*BP*, the EMG_t_, and the RCP. This is an important limitation as current models to describe the above mentioned breakpoints have linked the [HHb]*BP* and the RCP, at least in the VL muscle, to the maximal lactate steady state (MLSS) (Keir et al., 2015) which demarcates the upper boundary of prolonged sustainable performance (Mattioni Maturana et al., 2016). Identifying the association between changes in the profile of [HHb] and EMG signals with functional thresholds of performance in different muscles involved in cycling would provide important insights into the physiological mechanisms that control the limits of exercise tolerance.

Therefore, the aim of the present study was to investigate whether the [HHb]*BP*, normally observed toward the end of a RI cycling test in the VL, was also present in the VM and RF muscles. Furthermore, this study aimed to assess whether the occurrence of a breakpoint in the [HHb] profile in those specific muscles was also linked with the RCP and the EMG_t_. We hypothesized that, due to the similarities in the [HHb] profiles between the VL and VM, the [HHb]*BP* would only occur in these muscles but not in the RF, and, therefore we speculated that the [HHb]*BP* would coincide with the RCP and the EMG_t_ only in the VL and VM muscles.

## Materials and methods

### Participants

Twelve individuals (10 men and 2 women: 31.0 ± 8.1 year; 74.3 ± 10.4 kg; body mass index = 23.8 ± 2.8 Kg/m^2^) voluntarily participated in the study. All participants were trained individuals who regularly partake in endurance exercise programs (cycling constitutes a large portion of their training schedule). Training volume and frequency were similar across all of them (4 to 5 times per week—1.5 to 2 h per session). They were non-smokers, non-obese and not undergoing any medical treatment that could affect their cardiovascular response to exercise. All procedures were approved by the Conjoint Health Research Ethics Board of the University of Calgary and all participants gave their informed consent in accordance with the Declaration of Helsinki.

### Protocol

Participants visited the laboratory in two separate occasions, with 48 h in between, to perform RI tests on an electromagnetically-braked cycle ergometer (Velotron, Dynafit Pro, Racer Mate, Seattle, WA). They were already familiarized with the testing procedures as they were routinely involved in research projects in our laboratory. Regardless of this testing experience, if the peak power output (PO_peak_) achieved in the second testing session was more than 10 W lower or higher than the first one, participants were asked to report to the laboratory a third occasion to perform another RI test. Only the two RIs with the highest PO_peak_ were included in the analysis. There was no statistical difference between test 1 and test 2 for PO_peak_ (358 ± 47 vs. 359 ± 48 W) (*P* > 0.05). Three subjects reported to the lab a third occasion as the PO_peak_ in the first two visits differed by more than 10 W. The RI test protocol consisted of a baseline of 4 min at 50 W, followed by a 30 W·min^−1^ (1 W every 2 s) and 25 W·min^−1^ (1 W every 2.4 s) ramp increments for men and women, respectively, as previously described (Murias et al., 2013a). Participants were asked to maintain the same cycling cadence throughout the entire test (baseline + ramp) in a range between 80 and 90 revolutions per minute. The RI test was stopped when the participants were unable to maintain the targeted cadence despite strong verbal encouragement, or due to volitional exhaustion. PO_peak_ was determined as the highest power output achieved. Participants were not allowed to visualize the PO during the tests.

### Measurements

A metabolic cart (Quark CPET, Cosmed, Rome, Italy) was used to measure breath-by-breath pulmonary gas exchanges. Inspired and expired flow rates were measured continuously through a low dead space turbine which was calibrated beforehand with a syringe of a known volume. After being calibrated according to the manufacturer specifications, inspired and expired gases were analyzed for concentrations of O_2_ and CO_2_.

NIRS (Oxiplex TS™, ISS, Champaign, IL) was used to monitor local muscle deoxygenation ([HHb]) on the VL, VM, and RF. Briefly, the system was composed of two channels consisting of eight laser diodes (for each channel) operating at two wavelengths (λ = 690 and 828 nm, four at each wavelengths), which were pulsed in rapid succession, and a photomultiplier tube. The lightweight plastic NIRS probes (connected to laser diodes and a photomultiplier tube by optical fibers) consisted of two parallel rows of light-emitting fibers and one detector fiber bundle; the source–detector separations for this probe were 2.0, 2.5, 3.0, and 3.5 cm for both wavelengths. [HHb] VL signal was collected in both testing sessions whereas [HHb] VM and RF were separately collected in only one of the sessions in a randomized order. The NIRS probes were placed on the belly of the muscles of the right leg after the skin area was shaved and wiped. An optically dense, black vinyl sheet was used to avoid the intrusion of external light, and an elastic bandage as well as double-sided tape were used to secure in place the probes to minimize movement. The apparatus was calibrated on each testing day after a warm-up of at least 30 min as per manufacturer recommendations. Data were stored online at an output frequency of 2 Hz, and reduced to 1-s bins for all subsequent analyses within the present study. Before removing the probe, the VL area was marked to ensure the consistency of the placement for the following visit.

Simultaneously with the [HHb] recordings, the EMG signals of the VL, VM, and RF muscles were collected with an 8-channel recording system (Delsys Inc, Boston, MA) at a sampling rate of 1,000 Hz. Bipolar surface electrodes (41 × 20 × 5 mm) (DE-2.1, Delsys Inc.) were positioned on the same leg in close proximity of the NIRS probe after the skin area was carefully prepared. Hair was shaved off and the epidermal layer gently abraded and cleaned with an alcohol swab in order to minimize the impedance. Electrodes were connected to the acquisition apparatus (Power Lab, ADInstruments, Bella Vista, Australia) linked to a computer software (LabChart 8, ADInstruments).

### Data analysis

Breath-by-breath $\overset{\cdot }{\text{V}}$O_2_ data were individually analyzed as previously described (Lamarra et al., 1987): aberrant data points that were three standard deviation (*SD*) from the local mean were removed and then linearly interpolated to 1 s intervals. The second-by-second data were then time aligned so that time “zero” represented the onset of RI test. As previously described (Boone and Bourgois, 2012), the mean response time (MRT) was calculated on an individual basis using Origin software (Origin, Origin Lab, Northampton, MA) to account for the lag time in the increase in $\overset{\cdot }{\text{V}}$O_2_ after the onset of the ramp portion of the RI test. Briefly, a double linear model was fitted from baseline to the previously established gas exchange threshold (GET). The MRT corresponded to the time delay between the onset of the RI test (i.e., 240 s) and the intersection of the forward extrapolation of the baseline $\overset{\cdot }{\text{V}}$O_2_ (slope constrained to “zero”) and backwards extrapolation of the linear $\overset{\cdot }{\text{V}}$O_2_-time relationship from GET.

The RCP was determined by two independent exercise physiologists through visual inspection using standard ventilatory and gas exchange indices, as previously described (Beaver et al., 1986). Briefly, RCP corresponded to the second disproportional increase (i.e., second breakpoint) in the V_E_/$\overset{\cdot }{\text{V}}$O_2_ relationship, where the end-tidal PCO_2_ began to fall after a period of isocapnic buffering. Additionally, the relationship between V_E_/VCO_2_ against $\overset{\cdot }{\text{V}}$O_2_ was considered for confirmation of the RCP. In case of a disagreement of more than 150 mL·min^−1^ in the result, the physiologists would revaluate together the profiles until a consensus was reached. $\overset{\cdot }{\text{V}}$O_2max_ was defined as the highest $\overset{\cdot }{\text{V}}$O_2_ computed from a 20-s rolling average.

As previously described (Spencer et al., 2012), the [HHb]—time relationship for the VL and the VM was modeled with the following piece-wise “double-linear” model:

$$\begin{array}{l} f=\text{if }\left(\text{x}<BP,g\left(\text{x}\right),h\left(\text{x}\right)\right)\\ g\left(\text{x}\right)=i_{1}+\left(s_{1}\cdot \text{x}\right)\\ i_{2}=i_{1}+\left(s_{1}\cdot BP\right)\\ h\left(\text{x}\right)\;=\;i_{2}+\left[s_{2}\cdot \left(\text{x}-BP\right)\right]\\ \text{fit }f\text{ to y}, \end{array}$$

where *f* is the double-linear function, x is time and y is [HHb], *BP* is the time coordinate corresponding to the interception of the two regression lines (i.e., the [HHb]*BP*), *i*_1_ and *i*_2_ are the intercepts of the first and second linear function, respectively and *s*_1_ and *s*_2_ are the slopes. Model parameter estimates for each individual were determined by linear least-square regression analysis. The double linear fit was started at the onset of the systematic increase in the [HHb] signal until the last data point corresponding to the end of the test. Aberrant data that were 3 *SD* from the local mean were removed. The RF [HHb]*BP* profile was visually determined by two independent physiologists as, in the majority of the cases, the profiles of the [HHb] signal did not conform to a double-linear model fit. Specifically, the [HHb]*BP* was detected as the point at which the increase in the signal would change its slope (attenuation and/or steeper increase).

The EMG signals were amplified, rectified, band-pass filtered (5–500 Hz) and the root mean square (RMS) was calculated for every second. The averaged EMG profiles were then examined by two independent observers to detect the EMG_t_. The EMG_t_ was calculated as the second non-linear increase in RMS (Hug et al., 2003a,b). As cycling is a continuous of dynamic contractions, the RMS recorded during the last 2 min of 50 W 4-min baseline was used to normalize the ramp portion of the test.

The averaged and time-aligned [HHb] and EMG data were then plotted against the absolute $\overset{\cdot }{\text{V}}$O_2_ data. After being left-shifted according to the previously determined MRT, the $\overset{\cdot }{\text{V}}$O_2_ associated with the [HHb]*BP* and the EMG_t_ was found (Fontana et al., 2015).

For our analysis, the VL [HHb] and EMG signals of the two RI tests were averaged to elicit a single profile for the [HHb] and EMG, respectively. It has been demonstrated that both the [HHb] (Iannetta et al., 2017) and the EMG (Dorel et al., 2008) profiles of the VL during a RI test have a good inter-day repeatability.

The slopes of the increase in the [HHb] and EMG signals were calculated as follows: first, the data were normalized from 0 to 100%, 0% representing the baseline and 100% representing the peak value. The slope of the increase in both signals measured at 10% intervals was then calculated. This slope represents the rate of increase in each of the signals for a given percentage of increase in PO.

All statistics were performed using SPSS version 23 (SPSS, Chicago, IL). Descriptive data are presented as mean ± *SD*. Pearson's correlation coefficient was used to find correlation between variables. After ensuring the normal distribution of the data set (Shapiro-Wilk test), a paired-samples *t*-test (slope analysis) and a repeated-measure ANOVA (metabolic rates associated with RCP, [HHb]*BP*, and EMGt) were used to detect differences between the variables of interest. Statistical significance was set at a *P* < 0.05.

## Results

Average PO_peak_ of the RI tests were 359 ± 48 W. $\overset{\cdot }{\text{V}}$O_2max_ was 3.87 ± 0.46 L·min^−1^ (52.5 ± 6.0 mL·kg^−1^·min^−1^). The average MRT for the RI test was 32.9 ± 21.4 s. There was no difference between test 1 and test 2 for RCP (3.40 ± 0.41 L·min^−1^ vs. 3.38 ± 0.42 L·min^−1^) (*P* > 0.05). The absolute $\overset{\cdot }{\text{V}}$O_2_ (L·min^−1^) values corresponding to the RCP, [HHb]*BP*, and the EMG_t_ are shown in Table 1 along with the percent of $\overset{\cdot }{\text{V}}$O_2max_ and PO_peak_ at which these thresholds occurred. No significant differences were found between the $\overset{\cdot }{\text{V}}$O_2_ (L·min^−1^) at the RCP, the [HHb]*BP*, and the EMG_t_ for all three muscles (VL, VM, RF) (*P* > 0.05). Pearson's correlation index showed a significant association between the RCP, the [HHb]*BP*, and EMG_t_, with these correlations ranging from 0.90 to 0.97 for the muscles investigated (*P* < 0.05).

**Table 1:** $\overset{\cdot }{\text{V}}$O_2_ (L·min^−1^), percentage of $\overset{\cdot }{\text{V}}$O_2max_ and Peak Power Output (PO_peak_) associated with the respiratory compensation point (RCP), oxygen extraction breakpoint ([HHb]*BP*), and EMG threshold (EMG_t_).

		**Mean $\overset{\cdot }{\text{V}}$O_2_ ± *SD***	**%$\overset{\cdot }{\text{V}}$O_2max_ ±*SD***	**%PO_peak_ ± *SD***
	RCP	3.39 ± 0.41	87.5 ± 2.9	78.1 ± 6.8
Vastus lateralis	[HHb]*BP*	3.49 ± 0.46	90.1 ± 4.4	80.8 ± 5.5
	EMG_t_	3.40 ± 0.44	87.7 ± 4.6	78.3 ± 5.4
Vastus medialis	[HHb]*BP*	3.44 ± 0.61	88.6 ± 5.8	79.7 ± 3.7
	EMG_t_	3.43 ± 0.49	88.5 ± 5.7	79.1 ± 5.4
Rectus femoris	[HHb]*BP*	3.59 ± 0.52	92.4 ± 5.1	83.2 ± 4.7
	EMG_t_	3.48 ± 0.46	89.7 ± 5.5	80.2 ± 5.2

The amplitude of the [HHb] response in the RF (9.2 ± 7.8 μM) was significantly smaller than that of the VL (16.1 ± 11.1 μM) and VM (13.4 ± 8.7 μM) (*P* < 0.05). In the VL and VM muscles, the typical plateau-like response in the [HHb] signal was observed in all participants. This plateau-like response was found in only four subjects in the RF muscle. In the remaining eight participants, no plateau was observed in the RF muscle as the breakpoint in the [HHb] profile demarcated the onset of a steeper increase in the signal. No correlations were found between the [HHb] amplitude in each of the investigated muscles and the $\overset{\cdot }{\text{V}}$O_2max_ (either absolute and relative values) (*P* > 0.05).

**Figure 1:**
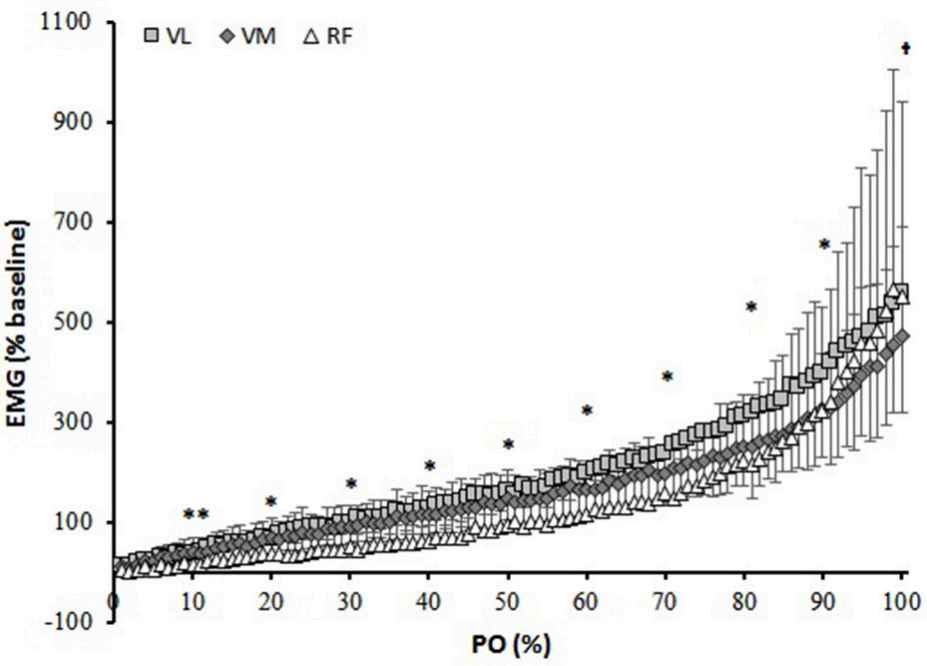
Muscle activation (Root mean square, RMS) normalized to baseline cycling over percentage power output (PO). ^**^different than VL and VM; ^*^all different from each other; ^†^different than VL and RF.

The RMS amplitude at the end of the RI test was 583 ± 178, 480 ± 214, and 583 ± 353% of baseline for VL, VM, and RF, respectively (Figure 1). The EMG_t_ was detected in all participants and within all muscles investigated. Except for the 0–10% PO range (*P* > 0.05), overall activation of the VL was significantly higher than that of the VM (*P* < 0.05). RF muscle activation was lower from VL and VM throughout the entire test (*P* < 0.05), except for the last 10% that was found to be similar to VL (*P* > 0.05).

**Figure 2:**
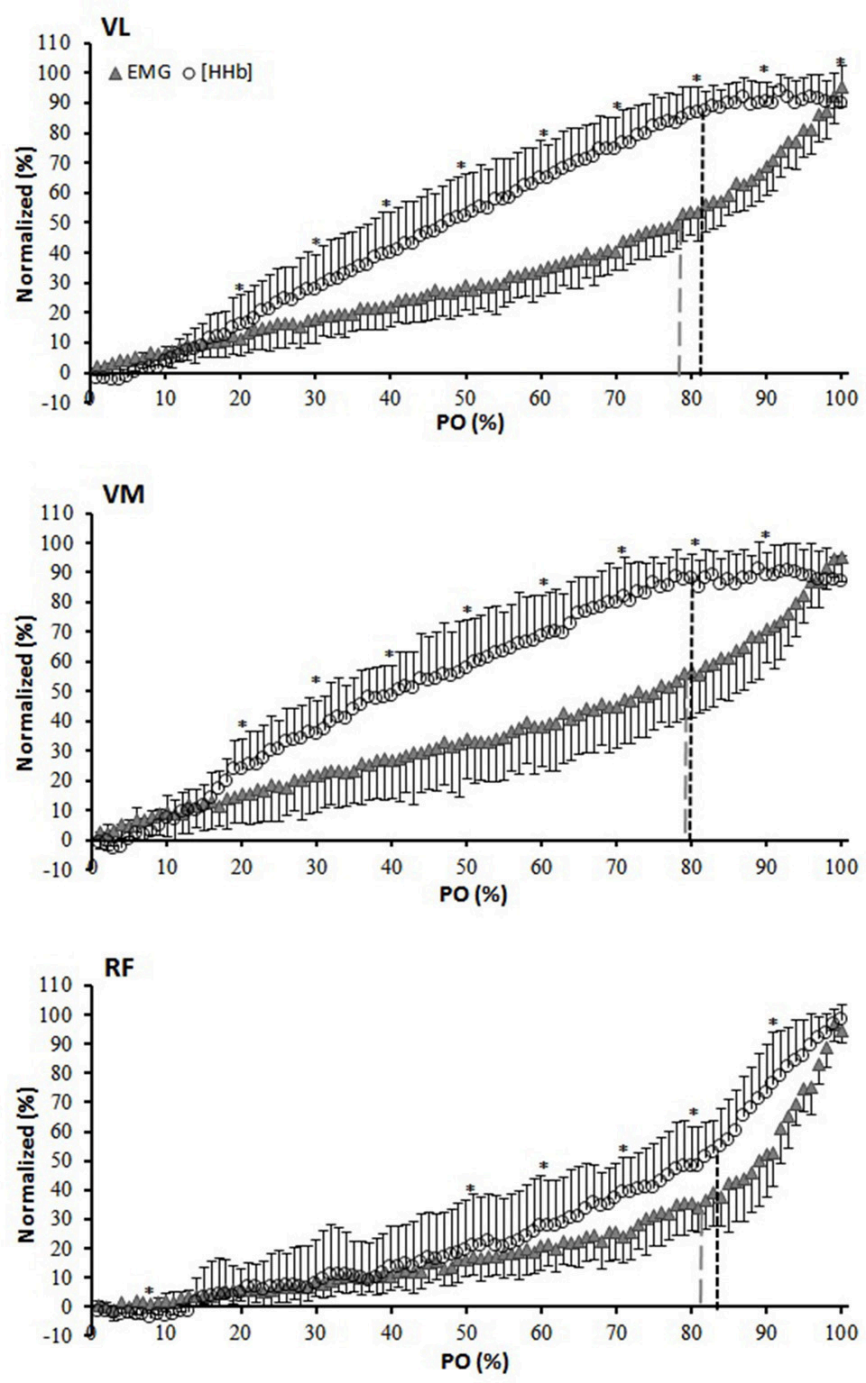
Normalized (0–100% of the amplitude) group average for [HHb] and EMG expressed as a percent of the normalized (0–100% of the response) power output (PO) for the vastus lateralis (VL), vastus medialis (VM), and rectus femoris (RF) muscles. ^*^[HHb] significantly different from EMG. Black and gray dashed lines indicate breakpoints in the [HHb] and EMG profiles, respectively.

**Figure 3:**
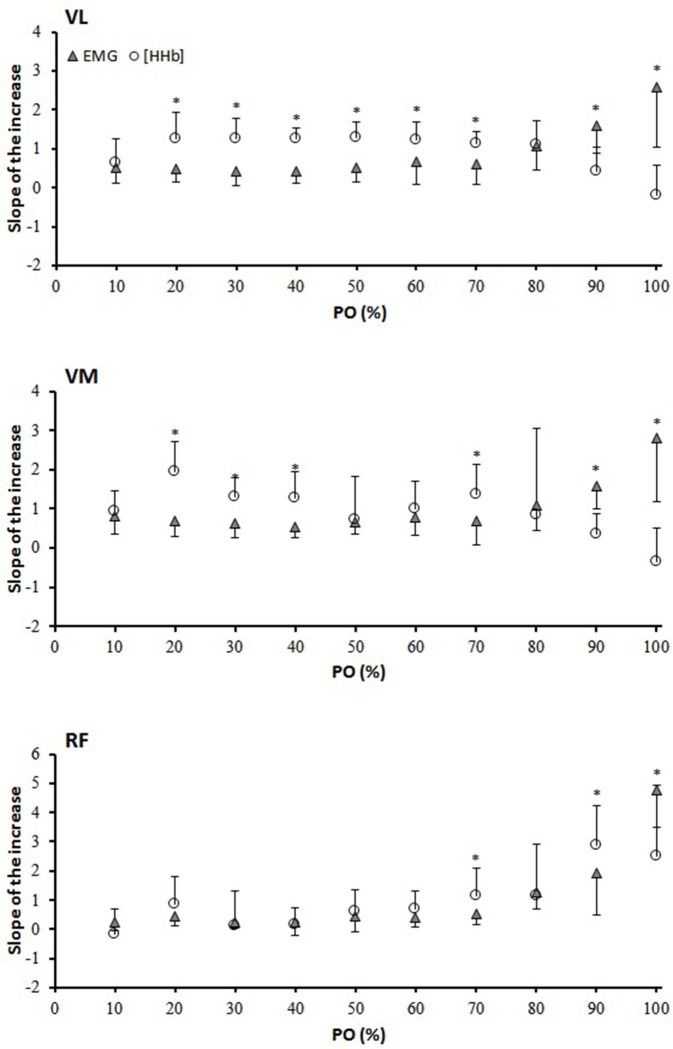
Slope analysis for the vastus lateralis (VL), vastus medialis (VM), and rectus femoris (RF) muscles. The slope of the increase and/or decrease was calculated between two data points every 10% interval of the PO. ^*^[HHb] significantly different from EMG.

Figure 2 depicts the percent change in [HHb] and EMG against the normalized PO_peak_ for the three muscles. For the VL muscle, the percent change in the [HHb] signal was significantly greater than that of the EMG from 20 to 100% of the response (*P* < 0.05). For VM, the [HHb] percent change was significantly greater than that of the EMG from 20–90% of the response (*P* < 0.05). For the RF muscle, the percent change in the [HHb] was significantly greater than that of the EMG signals from 50 to 100% of the response (*P* < 0.05). Figure 3 shows the differences in the slope of the increase in the [HHb] and EMG for VL, VM, and RF. In the 20–70% of PO_peak_ portion the average slope of the increase in the [HHb] signal was 1.21, 1.20, 0.67, and in the EMG signal was 0.59, 0.72, 0.51, in the VL, VM, and RF, respectively (*P* < 0.05). In the last portion of the test (80–100%) the slope of the increase in the [HHb] was 0.11, –0.01, 2.67 and in the EMG was 2.08, 2.18, 3.34 in VL, VM, and RF, respectively (*P* < 0.05).

## Discussion

Recent studies have established an association of the [HHb]*BP* as well as the EMG_t_ with the RCP in the VL during a RI test (Hug et al., 2003a; Murias et al., 2013b; Racinais et al., 2014; Fontana et al., 2015; Keir et al., 2015; Iannetta et al., 2017). However, this response has not been investigated in other active muscles during cycling exercise such as the VM and RF. Thus, the aim of the present investigation was to examine the association among the [HHb]*BP*, the EMG_t_, and the RCP not only in the VL but also in the VM, and RF muscles during a RI cycling test to exhaustion.

In line with previous studies (Hug et al., 2003a; Murias et al., 2013b; Racinais et al., 2014; Fontana et al., 2015; Keir et al., 2015; Iannetta et al., 2017) the present experiment found a correspondence between the [HHb]*BP* and the EMG_t_ with the RCP in the VL muscle. The novelty of the present investigation, however, is that this correspondence was also observed in the VM and in the RF muscles. Therefore, it seems that these physiological thresholds may share similar mechanistic basis and, as previously suggested (Keir et al., 2015), to reflect the boundary between the heavy and the severe intensity domains.

In regards of muscle activation, the profiles of the increase were very similar among the three investigated muscles showing the typical steeper increase at ~80% of the PO_peak_ (Figure 1). This disproportional increase in muscle activation that is identified as the EMG_t_ is likely due to the progressive recruitment of a greater number of motor units, in particular fast twitch motor units, that are needed to sustain the increasing workload and to compensate for the fatigue of the initially recruited fibers (Moritani et al., 1992; Scheuermann et al., 2002; Chin et al., 2011) (although an increase in discharge rate cannot be completely ruled out). In correspondence to the EMG_t_, the [HHb] signal also showed a breakpoint in all the muscles investigated. However, whereas in the VL and VM it led to a plateau-like response, in the RF it demarcated the onset of a steeper increase in the majority of the subjects (despite a similar fashion of increase in the EMG activity) (Figure 2). Chin et al. (2011) suggested that the reason for these different behaviors in the [HHb] profiles among the three muscles can be due to their different relative contribution during the RI test which would elicit different profiles of oxygen extraction. As showed in Figure 1, the RF activation was significantly lower virtually across the entire range of power outputs (from 0 to 90%) and, as a consequence, this might have produced a lower need to extract O_2_ as Q_m_, specifically in the RF muscle, would match the local $\overset{\cdot }{\text{V}}$O_2_. It is possible that due to biomechanical factors, the RF (a bi-articular muscle that during cycling assists with the hip flexion) is less activated when the resistance is low and becomes increasingly more involved when the “pushing” phase (mainly executed by VL and VM) has to be coupled by an enhanced “pulling” action (mainly executed by the RF) to sustain the increasing resistance (Jorge and Hull, 1986). However, this does not fully explain why the [HHb] signal plateaus in the VL and VM and increases further (at least in eight subjects) in the RF muscle in the presence of a common increase in muscle activation (as demonstrated by the EMG_t_). The causes of this difference might not be simply related to a temporal delayed activation of RF, but also to other factors, such as local vascular dynamic adjustments (Q_m_/$\overset{\cdot }{\text{V}}$O_2m_) that may be related to different fiber type distribution among these muscles. The RF has been shown to have a lower proportion of type I fibers (~30%) (Johnson et al., 1973) than VL (~40–50%) (Johnson et al., 1973; Edgerton et al., 1975; Green et al., 1979; Staron et al., 2000), and VM (~50%) (Johnson et al., 1973). It has been demonstrated that slow twitch fibers have a greater number of capillaries around each fiber (higher capillary-to-fiber ratio) (Andersen, 1975) and show a better vasodilatory dynamic control in comparison to fast twitch fibers (Wunsch et al., 2000; McDonough et al., 2005; Ferreira et al., 2006). Therefore, as metabolites (i.e., lactate, K^+^, H^+^) accumulate within and in proximity to the active tissues with increasing intensity of exertion, these vasoactive signals might trigger an increased relaxation of smooth muscles in the vessels near these highly active fibers causing the redistribution of the blood flow from the areas with less O_2_ demand toward those with higher O_2_ demand. This would produce toward the end of a RI test, particularly in the VL and VM muscles but not in the RF muscle, the plateau in the [HHb] signal, which, would therefore be the result of a greater amount of blood supplying the active muscle fibers. The fact that in four subjects the breakpoint in the RF [HHb] led to a plateau whereas in eight it represented the onset of a steeper increase in the signal demonstrates that, rather than only differences in activation, differences in oxygen extraction capacity and vascular dynamic controls are responsible for the heterogeneity in the [HHb] profiles within these muscles (Heinonen et al., 2015). In support to this, Okushima et al. (2015) found that differences in the oxygen extraction capacity exists even between deep (more oxidative) and superficial (less oxidative) regions of the quadriceps muscle which may be due to different characteristics (in terms of vascular control) of different muscular compartments.

It is important to acknowledge that at near-maximal intensity of exercise there might exist an enhanced competition for Q between locomotor muscles and respiratory muscles (e.g., diaphragm, intercostals), with a greater amount of blood redirected toward the latter rather than to the former, as previously suggested (Harms et al., 1998). However, Calbet et al. (2007) have shown that during a combined leg and arm incremental exercise $\overset{\cdot }{\text{Q}}$, O_2_ delivery, and vascular conductance continued to increase until exhaustion in the legs whereas in the arms as well as in the head and trunk either decreased or remained unchanged.

As depicted in Figure 3, the [HHb] signal in the VL and in the VM muscles increased at a faster rate (from ~10 to ~80% of PPO) than that of the RF when comparing the slope of the increases in the EMG signal (despite the better vascularization that is expected in type I fibers (McDonough et al., 2005 compared to the more anaerobic type II fibers). This could be partly related to different patterns of muscle activation previously described but also to the fact that, as slow twitch fibers are generally accompanied by a higher concentration of enzymes involved in the oxidative process, a greater extraction potential is expected within those fibers (Saltin et al., 1977; Delp and Duan, 1996). Therefore, a similar increase in muscle activation may induce a greater extraction signal in type I fibers (e.g., VL and VM) compared to type II fibers, even though vascular adjustments are supposedly to be improved in the former than in the latter. In support of this, in our study the amplitude in the [HHb] signal was greater in VL and VM than in RF despite similar activation (EMG) at peak exercise. In contrast to the observations of Chin et al. (2011), and other authors (Hug et al., 2006) the present study did not find any difference in muscle activation between VL and RF at peak exercise (with VM being lower). It could be speculated that, at least at maximal intensity, the contribution of the three muscles investigated was similar (despite the different temporal activation during the pedaling motion) and therefore the differences in O_2_ extraction (VL and VM > RF) might be explained by different O_2_ extraction capacity and/or need. In the study of Chin et al. (2011) the EMG signal was normalized against an isometric maximal voluntary contraction (MVC). While this approach is considered valid for static muscular actions, underestimations of the total contribution of the RF compared to VL and VM are possible during dynamic contractions (Hunter et al., 2002; Burden, 2010). In the present investigation, the normalization of the RMS during the RI test was made against the RMS recorded during the last 2 min of baseline cycling, which represents a more specific and reproducible approach than normalizing against an MVC.

In an effort to sequence the physiological events related to exercise intensity boundaries during RI tests, Boone et al. (2016) found in a large and heterogeneous sample, that within a 2% range (~86–88% $\overset{\cdot }{\text{V}}$O_2max_) the EMG_t_ significantly preceded the RCP, which in turn significantly preceded the [HHb]*BP*. However, in that study, when the comparison is made in terms of absolute $\overset{\cdot }{\text{V}}$O_2_ at which the thresholds occurred, the RCP and the [HHb]*BP* are not significantly different, and the differences in $\overset{\cdot }{\text{V}}$O_2_ against the EMG_t_ are only 52 and 83 mL·min^−1^, respectively. These differences in $\overset{\cdot }{\text{V}}$O_2_ are extremely small, and, in our interpretation, unlikely to be physiologically meaningful, especially if one considers that the average $\overset{\cdot }{\text{V}}$O_2_ fluctuation (noise) during cycling at (only) 90% of the GET has been measured to be 139 mL·min^−1^ (Keir et al., 2014). Therefore, although these boundaries of exercise intensity can be measured from systemic (RCP) and local ([HHb]*BP* and EMG_t_) signals, what is important is that all these signals seem to reflect similar metabolic thresholds intensities and that, even if small differences in the percent $\overset{\cdot }{\text{V}}$O_2_ associated with these thresholds occur (which were not significantly different in the present study), those small differences might simply reflect alterations related to the site and tool of measurement as well as the inspection strategy that was used to estimate the different thresholds. In this perspective future investigations should focus on comparing the differences in terms of accuracy and reliability of the devices that were used by different research laboratories to detect these important functional thresholds.

## Limitations

The analysis of the [HHb] and EMG slopes was performed to describe the rate of increase and decrease in these two signals. Although useful to individually interpret the changes in each of these signals, direct comparisons between changes in the slope of the [HHb] and EMG signals should be interpreted with caution as they originate from different tools with different properties for data collection. Furthermore, although in the present study the EMG_t_ was clearly displayed by all the participants in all the muscles investigated (VL, VM, RF), the idea of an EMGt should be interpreted with caution as some studies have showed that, for example, in some participants and/or muscles this functional threshold may not occur and/or not be evident.

In addition, even if the way EMG data were normalized in the present study is probably the most appropriate one, one cannot ascertain whether it reflects the true motor unit activation. In addition, any increase of the discharge rate beyond the fusion frequency will increase EMG but will not be translated in force production. Finally, the action potentials amplitude is higher in type II fibers so that their recruitment affects the EMG signal to a higher extent compared to the recruitment of type I fibers.

## Conclusions

In summary, the present study is the first to demonstrated that when a certain metabolic rate is achieved (~80–90% $\overset{\cdot }{\text{V}}$O_2max_), local and systemic physiological responses are manifested. Indeed, it was shown that the metabolic rate at the RCP was not different than that at the [HHb]*BP* and the EMG_t_. However, the [HHb]*BP* led to a plateau of the response in the VL and VM, in contrast to a steeper increase of the signal in the majority of the subjects in the RF. These findings reinforce the idea that regional heterogeneities in oxygen extraction exist which may be due not only to different patterns of muscle activation, but also to the interaction of regional differences in oxygen provision and oxygen extraction capacity. Although this is a novel and important aspect that needed to be studied to better interpret physiological responses to incremental exercise to exhaustion within the active tissues, future investigations should consider to study longitudinal changes (e.g., due to training, or detraining) in order to evaluate if the correspondence among these thresholds remains consistent. In addition, it would be worth to investigate also other exercise modalities (e.g., running) in order to compare the responses between mono- and bi-articular muscles. Overall, this will be of great importance as it would help researchers to distinguish whether the relationship between these thresholds is a mere coincidence or it is in fact due to shared mechanistic basis.

## Author contributions

DI, AQ, and JM contributed to the conception of the work, and the data acquisition. DI, GM, and JM interpreted the data. DI wrote the first draft of the manuscript. All the authors revised and approved the manuscript. In addition, they agreed to be accountable for all aspects of the work in ensuring that questions related to the accuracy or integrity of any part of the work are appropriately investigated and resolved.

### Conflict of interest statement

The authors declare that the research was conducted in the absence of any commercial or financial relationships that could be construed as a potential conflict of interest.
